# Application of Phosphate-Based Binders for the Stabilization and Solidification of Metal-Contaminated Soil: Mechanisms and Efficacy Evaluation

**DOI:** 10.3390/toxics12120907

**Published:** 2024-12-13

**Authors:** Shiliang Xu, Ayesha Imtiyaz Cheema, Yunhui Zhang, Bin Dong

**Affiliations:** 1 College of Environmental Science and Engineering, Tongji University, Siping Road, Shanghai 200092, China; 2 Shanghai Municipal Engineering Design Institute (Group) Co., Ltd., Shanghai 200437, China; 3 School of Architecture and Civil Engineering, West Anhui University, Lu’an 237012, China

**Keywords:** stabilization/solidification, multi-metal contamination, phosphate binders, ordinary Portland cement, leachates

## Abstract

At present, contamination due to toxic metals is a global concern. The management of problems caused by heavy metals relies on stabilization/solidification, which is the most effective technique for the control of metal pollution in soil. This study examined the immobilization efficiency of various phosphate-based binders (Na_3_PO_4_, Na_2_HPO_4_, NaH_2_PO_4_), in addition to ordinary Portland cement (OPC), MgO, and CaO, for the stabilization of multi-metal-contaminated soils. Moreover, this study focused on the leachability of copper, nickel, zinc, lead, cadmium, and manganese (Cu, Ni, Zn, Pb, Cd, Mn, respectively) over different time periods and with different concentrations. Batch leaching experiments were conducted to determine the leaching ratios and percentages of the various metal concentrations, along with measuring the pH values of the leachates. Our results indicate that the use of OPC was validated due to its superior immobilization performance across all metals present in the soil, but particularly with regard to metals in high concentrations. This was due to the formation of stable hydroxides and the high pH values, which assisted in abating the metals’ solubility. Additionally, phosphate-based binders, despite being environmentally favorable, were found to be less effective, particularly for Pb and Cu, and the leaching results exceeded non-hazardous waste limits. MgO showed reasonable immobilization results but was less effective compared to OPC; on the other hand, CaO exhibited increased leaching over time. Therefore, the present research serves primarily to highlight that OPC is more suitable for soil remediation at industrial sites and in the construction of infrastructure. Meanwhile, phosphate-based binders are shown to be more appropriate for eco-friendly, non-load-bearing applications.

## 1. Introduction

Soil contamination and the serious environmental issues caused by heavy metals are a major environmental concern globally. These environmental issues not only concern agricultural ecosystems but also affect other environmental components. Waste from several industries and other anthropogenic activities—such as mining, smelting, the manufacturing of new products, and the improper discarding of hazardous toxic waste and effluents [1]—has led to the extensive distribution of toxic metals such as copper (Cu), lead (Pb), manganese (Mn), zinc (Zn), nickel (Ni), and cadmium (Cd) in soils [2].

The transportation of these metals is predominantly caused by post-mining processes, which include soil erosion, surface run-off, and underground run-off over long distances. These toxic metals pose a serious risk to ecosystems as well as human health due to their ability to persist in the environment, their continuity, their potential bioaccumulation, and toxicity issues [3]. Therefore, remediation strategies are being developed to control this pollution on a commercial scale. For example, in order to control metal pollution, the in situ stabilization and solidification (S/S) technique, also known as the “green technique”, transforms noxious and hazardous waste into a thermodynamically stable material [4]. This method has been found to be a cost-effective, economical, and environmentally friendly solution to not only immobilize contaminants but also reduce their bioavailability in contaminated soils [5]. This method is more effective and has more practical implications compared to other traditional remediation methods, such as excavation/digging and the off-site disposal of waste, both of which are costly and impractical for large-scale commercial applications [6]. Moreover, the stabilization/solidification technique involves various chemical binders being placed into contaminated soil in order to reduce the toxicity of the pollutants, relying on the physical encapsulation method (solidification) or chemical transformation (stabilization) [6]. This technique not only mitigates and immobilizes pollutants but also improves the soil structure.

According to previous studies, these novel binders are capable of stabilizing metals and their occurrence in various forms, which ensures the long-term durability of the soil and enhances its mechanical strength under different environmental conditions [7]. The effectiveness of the most globally known binder, ordinary Portland cement (OPC), has been proven due to its ability to immobilize heavy metals [8] via a hydration reaction mechanism that further results in stable compounds [9]. However, substitute binders have been proposed due to OPC’s environmental impact, particularly its high carbon dioxide emissions during production and the potential leaching of various metals over time. Production of one ton of ordinary Portland cement requires 80–110 kW⋅h of electricity and emits 1.01 tons of carbon dioxide [10]. In order to sequester the carbon dioxide emitted when producing calcium and magnesium carbonates [11], magnesium oxide (MgO) and calcium oxide (CaO) are applied as binders for soil stabilization [12]; they also have the tendency to create insoluble metal hydroxides when they react with pollutants and soil moisture. In addition, these binders offer certain benefits, although their effectiveness may be restricted by multiple factors, such as maintaining the pH and the establishment of reactions that create products that can compromise the soil’s integrity [13]. Furthermore, the compressive strength plays a crucial role in the strengthening of the material, enabling it to withstand greater pressure, as measured in multi-metal-polluted soils treated with different phosphate-based binders and additives, including OPC, MgO, CaO, sodium phosphate (Na_3_PO_4_), monosodium phosphate (NaH_2_PO_4_), and disodium phosphate (Na_2_HPO_4_).

In consideration of these factors, the phosphate-based binders Na_3_PO_4_, NaH_2_PO_4_, and Na_2_HPO_4_ are indicated as the most promising substitutes due to their ability to produce stable, less soluble metal–phosphate compounds [14]. This is also due to their ability to immobilize and leach toxic metals such as Pb, Zn, Mn, Cd, Ni, and Mn [15] by creating stable compounds such as pyromorphite (Pb_5_(PO_4_)_3_Cl), which is resistant to dissolution under acidic environments. Several studies have focused on the stabilization of metals or a particular metal group, but phosphate binders, despite their potential efficiency, have been less explored in scientific research. These binders have substantial long-term effects on various environmental aspects, including variations in pH, fluctuations among microbial communities, and the moisture content [16]. However, there is limited knowledge related to the efficiency of phosphate compounds in stabilizing multi-metal systems.

Thus, this study mainly focuses on addressing (1) the influence of different phosphate-based binders (Na_3_PO_4_, Na_2_HPO_4_, NaH_2_PO_4_) on the leachate pH and the compressive strength of multi-metal-contaminated soils; (2) to assess the efficiency of phosphate-based binders in immobilizing heavy metals by estimating the stabilization/solidification activity, focusing on Pb, Cd, Zn, and Cu in comparison to traditional binders such as OPC, MgO, and CaO; and (3) to study the long-term stability and leachability of metals from treated soils under various environmental conditions. Through this research, we aim to contribute to the development of effective, sustainable, and economically viable strategies for the management of heavy metal contamination in soils, thereby contributing to the protection of human health and the environment.

## 2. Materials and Methods

### 2.1. Material Collection

Kaolin and sand were used to represent sandy soil. Artificial multi-metal soil contamination was achieved with zinc nitrate hexahydrate (Zn (NO_3_)_2_·6H_2_O), manganese (II) nitrate hexahydrate (Mn (NO_3_)_2_·6H_2_O), cadmium nitrate tetrahydrate (Cd (NO_3_)_2_·4H_2_O), nickel (II) nitrate hexahydrate (Ni (NO_3_)_2_·6H_2_O), copper (II) nitrate trihydrate (Cu (NO_3_)_2_·3H_2_O), and lead (II) and lead (II) nitrate (Pb (NO_3_)_2_) compounds with a concentration of 1000 mg/kg or 2000 mg/kg, as well as nickel (II) nitrate hexahydrate (Ni(NO_3_)_2_.6H_2_O), copper(II) nitrate trihydrate (Cu(NO_3_)_2_·3H_2_O), and lead(II) nitrate (Pb(NO_3_)_2_) with a concentration of 1000 mg/kg or 2000 mg/kg. Distilled water was used to produce solutions to carry out the analysis.

Three phosphate salts (Na_3_PO_4_, Na_2_HPO_4_, and NaH_2_PO_4_) and two alkali additives (MgO and CaO) were used as binders, and their S/S performance for the various metal-contaminated soils was also compared with that of ordinary Portland cement. The OPC (CEM I 52.5 R) was sourced from Buildmate Pte Ltd., located in Singapore. Meishen Chemical Co. Ltd., Xingtai, China, provided MgO containing 46-seconds reactivity [17]. Furthermore, we purchased CaO (purity grade) from Sigma-Aldrich Pte Ltd. in Singapore. The physicochemical properties of the kaolin and OPC were in line with [18]. The sand-to-kaolin ratio was established at 9:1. In order to protect the samples from contamination, and before the artificial dry soil was placed into split PVC molds (100 mm in height and 50 mm in diameter), it was thoroughly mixed with 5% of the six additives and 7.5% water. After this, the specimens were sealed in plastic bags and allowed to cure for 7 and 28 days at 25 °C and 95% humidity in a storage chamber.

### 2.2. Experimental Methodology

After curing, the samples were demolded and their mechanical strength was measured using the unconfined compressive strength test, which was conducted with two instruments (ELE digital tritest and Wykeham Farrance WF10026 TRITECH10, with a maximum capacity of 20 kN and 10 kN, respectively) at a constant rate of 1.0 mm/min. Experiments were performed in triplicate considering quality assurance. After this, the batch leaching tests were conducted according to [19]. For further specific analyses of the crushed freeze-dried samples, specimens with particle sizes of less than 4 mm were mixed with 900 mL distilled water in a 2 L polypropylene plastic bottle. Later, the mixture’s pH was determined using a pH instrument (Mettler Toledo Sevencompact S220) after it was stirred for 24 h at a speed of 8 revolutions per minute on a VELP Scientifica Overhead Mixer Rotax 6.8. The mixture was then filtered using a 0.45 μm membrane syringe filter and the total metal concentration was determined using a PerkinElmer Elan DRC-e inductively coupled plasma mass spectrometry (ICP-MS) instrument.

It should be noted that the threshold for metal concentration characterization using ICP-MS is one part per billion (ppb). The experimental procedures were performed in triplicate, and blank experiments were undertaken under the same conditions as described in our previous study [20]. In addition, the residual crushed samples were desiccated using a freezing vacuum drier and then pulverized to achieve a particle size below 0.075 mm for the study of their microstructures. This included X-ray diffraction (XRD), Fourier transform infrared spectroscopy (FTIR), and thermogravimetric analysis (TGA). X-ray diffraction (XRD) was conducted using a Bruker D8 X-ray diffractometer equipped with a Cu Kα source operating at 40 kV and 40 mA. The TGA experiment was performed with the PerkinElmer TGA 4000 instrument, heating the sample from 30 to 900 °C at a rate of 10°/min in a N_2_ atmosphere.

#### Statistical Analysis

The data derived from characterization were statistically analyzed using the Origin software 2024 (Origin Lab Corp., Northampton, MA, USA). The data presented in the graphs regarding the metal concentrations are reported as the mean and standard deviation of three replicates for each treatment; the graphs were created using Microsoft PowerPoint, 2021 version 2108 (Build 14332.20812), and Origin software 2024 (OriginLab Corp., Northampton, MA, USA). To ensure data accuracy and quality assurance, we repeated the test in three replications and used distilled water to perform the experiment. Before the artificial dry soil was placed into split PVC molds (100 mm in height and 50 mm in diameter), it was thoroughly mixed with 5% of the six additives and 7.5% water. After this, the specimens were sealed in plastic bags and allowed to cure for 7 and 28 days at 25 °C and 95% humidity in a storage chamber.

## 3. Results and Discussion

### 3.1. Unconfined Compressive Strength

**Figure 1:**
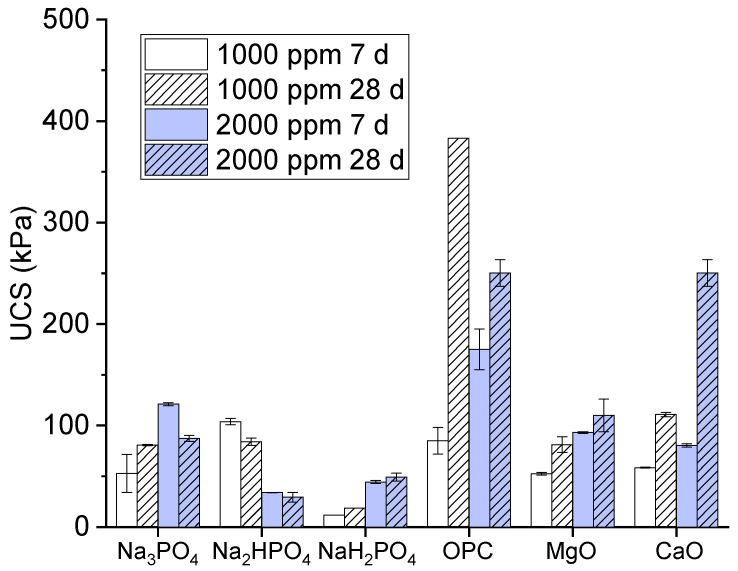
Unconfined compressive strength values of contaminated soils cured for 7 days and 28 days.

The unconfined compressive strength is as a measure of the load-bearing capacity of a material (often a soil or rock) when it is subjected to compression without any lateral support. In the present study, we assessed the strength and stability of soils, particularly when they were subjected to a load or pressure. A high unconfined compressive strength value indicates a stronger material that can withstand greater pressure before failure. The unconfined compressive strength values of the multi-metal-polluted soils treated with different phosphate-based binders and additives, including ordinary Portland cement, MgO, and CaO, are depicted in Figure 1. The values of the unconfined compressive strength are expressed in kilopascals (kPa). The treatments consisted of two distinct concentrations (1000 parts per million and 2000 parts per million) and two different curing durations (7 days and 28 days).

The results of the present study regarding binders consisting of monosodium phosphate, disodium phosphate, and trisodium phosphate are explained as follows. Monosodium phosphate had the lowest unconfined compressive strength values among the polyphosphate-based binders. Meanwhile, Na_2_HPO_4_ exhibited lower water content saturation at its unconfined compressive strength values in comparison to Na_3_PO_4_. A clear correlation was found between a higher concentration and longer curing time and an increase in unconfined compressive strength, although the values remained somewhat uncertain. Moreover, the compound trisodium phosphate had intermediate unconfined compressive strength values, with greater strength recorded at 2000 ppm compared to 1000 ppm for curing periods of 7 and 28 days. Meanwhile, its strength rose proportionally with an extended curing duration.

Similarly, in the comparison of OPC, MgO, and CaO, the results showed that ordinary Portland cement exhibited the highest unconfined compressive strength values. These values exhibited a notable increase at 2000 ppm after 28 days of curing, reaching approximately 500 kPa. These findings suggest that ordinary Portland cement is highly efficient in enhancing the structural integrity of polluted soil. Moreover, magnesium oxide had moderate unconfined compressive strength values, being superior to CaO, Na_2_HPO_4_, and NaH_2_PO_4_, but inferior to Na_3_PO_4_ and OPC [21]. However, its strength rose proportionally with both an increased concentration and extended curing duration. Meanwhile, the unconfined compressive strength values of CaO are often lower than those obtained with OPC [22].

Next, we examine the reasons for the effectiveness of the phosphate-based binders, namely MgO and CaO, and their implications for the stabilization of contaminated soil. OPC efficiently stabilized the soil due to its hydraulic properties and pozzolanic reactions. A high binding strength was provided by calcium hydroxide and calcium silicate hydrate (C-S-H). These are formed when OPC is hydrated, creating a gel, and this material helps to bind the cement particles by filling the pore spaces and absorbing toxic metals; this is consistent with the results in [10]. Moreover, the C-S-H gel shows an absorption peak near 980 cm^−1^, representing Si-O vibrations, as also confirmed in [23,24]. Therefore, according to its high unconfined compressive strength values, OPC has the ability to encapsulate metal pollutants, which could render them less mobile and less likely to leach [25].

Additionally, compared to Na_2_HPO_4_ and NaH_2_PO_4_, Na_3_PO_4_ was the most effective phosphate-based binder, exhibiting the ability to raise the unconfined compressive strength. Na_3_PO_4_ may also facilitate stabilization through the formation of more stable complexes or precipitates, formed via chemical interactions with metal impurities. Since phosphate-based binders are less powerful than OPC, they may be insufficient to significantly increase the strength, but they could be useful in immobilizing some contaminants because of their capacity to react with water and produce binding phases with certain oxides (such as Mg (OH)_2_ and Ca (OH)_2_) [26].

Due to their moderate unconfined compressive strength values, we found that the MgO and CaO binders functioned well as binding agents; when combined with water, they generated Mg(OH)_2_ and Ca(OH)_2_. This allowed them to boost the strength by filling voids and cementing the soil particles together. These hydroxides also increase the pH, promoting the precipitation of metal hydroxides, which aids in contaminant stabilization [27]. Due to its sluggish hydration process, MgO is renowned for its ability to cause a modest but steady increase in strength. In contrast, calcium oxide has a faster reaction time in the production of calcium hydroxide, which helps to boost the initial strength [28]. Although their unconfined compressive strength and effectiveness are lower than those of OPC, these binders can nevertheless contribute to soil stabilization, particularly when mixed with other binders. The use of additional binders may accelerate chemical reactions, making the soil matrices denser and stronger [29]. However, increasing the binder concentration may raise the treatment costs and environmental impacts, such as carbon emissions due to OPC.

Moreover, the strength of the soil samples increased significantly with an extended curing period, from 7 days to 28 days. Longer curing periods facilitate the formation of more stable and crystalline structures, enhancing the unconfined compressive strength. For OPC, extended curing allowed more C-S-H to form, while, for the phosphate-based binders, longer curing could allow more complete complexation reactions. This observation underscores the importance of allowing adequate time for chemical stabilization processes to occur in remediation projects.

### 3.2. Leachability

**Figure 2:**
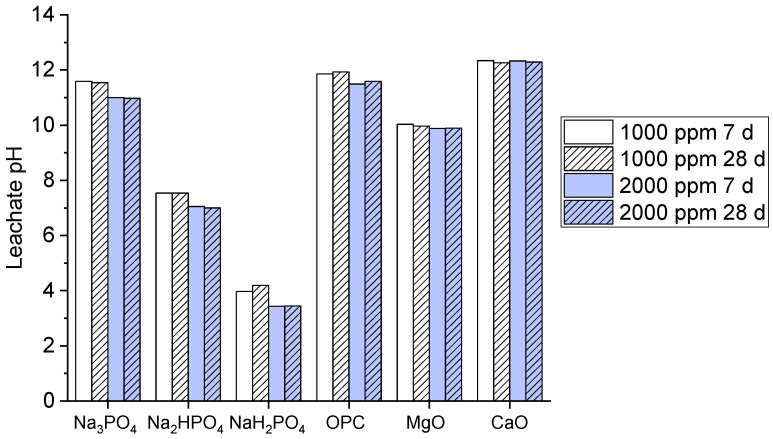
Leachate pH values of contaminated soils treated for 7 days and 28 days.

Leachability is a key factor enabling potential contaminants to be released into the environment when visible to other solvents. Typically, a high pH causes a reduction in leaching when used with other phosphate binders. The leachate pH values for the multi-metal-contaminated soils treated with the phosphate-based binders and other common binders (OPC, MgO, CaO) at concentrations of 1000 ppm and 2000 ppm and for curing periods of 7 and 28 are shown in Figure 2.

The graph shows that both Na_3_PO_4_ and Na_2_HPO_4_ exhibited a high pH value, approximately above 10, which indicates strong alkalinity, but the pH became high at 28 days at a 2000 ppm concentration. Meanwhile, NaH_2_PO_4_ demonstrated significantly lower pH values of 4–5, signifying its acidic nature. The OPC, MgO, and CaO binders maintained high pH values above nine, which reflect their alkaline nature, as compared to OPC and CaO, which presented the highest pH stability. The high pH values of Na_3_PO_4_ and Na_2_HPO_4_ may explain their effectiveness and efficiency in immobilizing metals via precipitation under alkaline conditions; this further enhances the stabilization of multi-metal contaminants [30]. However, the acidic nature of NaH_2_PO_4_ may explain its limited metal immobilization potential as compared to the other binders. Moreover, OPC, MgO, and CaO maintain strong alkalinity [31], which is typical for these binders; this supports their known capabilities in metal stabilization by raising the pH.

#### Leaching Concentrations of Metals in Contaminated Soils After 7 and 28 Days

**Figure 3:**
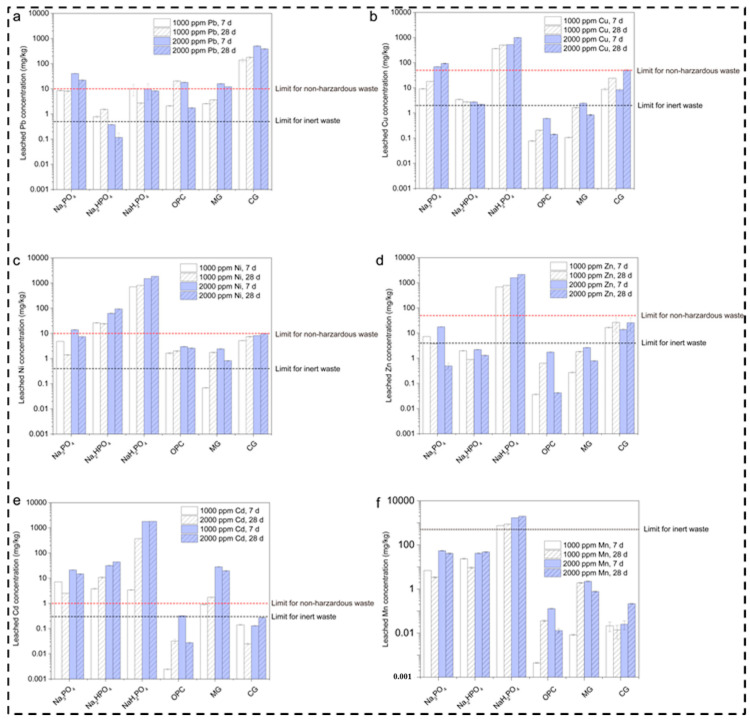
Leaching concentrations of metals in contaminated soils after 7 and 28 days. (**a**) Leached Pb concentration for various phosphate based binders (**b**) Leached Cu concentration for various phosphate based binders (**c**) Leached Ni concentration for various phosphate based binders (**d**) Leached Zn concentration for various phosphate based binders (**e**) Leached Cd concentration for various phosphate based binders (**f**) Leached Mn concentration for various phosphate based binders.

The leached lead concentrations in the multi-metal-contaminated soils treated with several binders (Na_3_PO_4_, Na_2_HPO_4_, NaH_2_PO_4_, OPC, MgO, and CaO) at concentrations of 1000 and 2000 ppm and at curing durations of 7 and 28 days are shown in Figure 3a. The comparison also considered the regulatory limits for non-hazardous and inert waste. The graph shows that Na_3_PO_4_ somewhat reduced the leached Pb concentration with the passage of time within the range of 1000–2000 ppm. Meanwhile, (Na_2_HPO_4_ + NaH_2_PO_4_), OPC, and MgO increased the reduction in the Pb concentration below the waste limit as compared to OPC and MgO, which moderately reduced the Pb concentration. This is consistent with the results reported in [32]. The reason behind the high Pb immobilization potential of NaH_2_PO_4_ is its acidic nature, which promotes metal bonding and the formation of stable complexes; this is inconsistent with [14]. Meanwhile, Pb is effectively reduced by Na_3_PO_4_ and Na_2_HPO_4_, although the concentration remains above the inert waste limit. Moreover, OPC and MgO exhibit temporary stabilization owing to their pH-buffering abilities, while CaO’s high leachability leads to poor Pb stabilization under these conditions.

Additionally, the graph shown in Figure 3b presents the leached copper concentration for various compounds, including Na_3_PO_4_, Na_2_HPO_4_, NaH_2_PO_4_, OPC, MgO, and CaO, at concentrations of 1000–2000 ppm over a period of 7 and 28 days. The results reveal that, among the different sodium phosphate compounds, Na_3_PO_4_ and Na_2_HPO_4_ enabled high Cu leaching when it surpassed the limit for non-hazardous waste, namely 100 mg/kg. This is due to the phosphate compounds, which form many insoluble metal–phosphate complexes, assisting in stabilization; this is also explained in [33,34]. According to our results, the mechanism of phosphorous involves the generation of less stable and soluble copper phosphate complexes, which vary due to the significant variations in the degree of dissociation in water. This reduces its ability to bind with copper ions, as explained in [35]. Likewise, MgO and CaO also demonstrated high leaching over time and with different concentrations, whereas MgO showed a moderate effect on Cu leaching, mostly below the hazardous limit [25]. Meanwhile, CaO slightly exceeded the limit at 2000 ppm and 28 days. This is because it has the ability to increase the pH, which leads to the precipitation of copper hydroxide layers with the capability to bind metals such as copper on their surfaces.

Similarly, for nickel, the leaching degree is relatively high at 2000 ppm after 28 days, showing the highest leaching, as described in Figure 3c. OPC, on the other hand, presents a decline in leaching at 2000 ppm after 28 days. Meanwhile, MgO and CaO exhibit moderate levels and surpass the inert waste limit after 28 days. The reason behind these results for the phosphate compounds is the same as for cadmium, namely the formation of highly insoluble complexes, which leads to significant leaching [36]. Similar behavior was observed for OPC, which lowered the Ni concentration as the cementitious matrix created a physical barrier around the Ni ions. Furthermore, OPC has a high pH; it is less soluble and can immobilize Ni. Additionally, MgO and CaO show moderate Ni leaching due to their ability to create an alkaline environment, which aids in Ni hydroxide precipitation, although it was diminished slightly at 28 days. This may be due to carbonation, which reduces the pH and destabilizes Ni compounds, making them more soluble [37].

Regarding zinc leaching, the results of the present study indicate that Na_3_PO_4_ and NaH_2_PO_4_ led to particularly high leaching at 2000 ppm after 28 days, as shown in Figure 3d. Meanwhile, Na_2_HPO_4_ showed lower zinc leaching as compared to the other phosphate compounds, although it still exceeded the inert waste limit of 2 mg/kg at both concentrations. OPC also showed a lower leaching level after 28 days, which indicates strong immobilization. However, MgO and CaO exhibited relatively high leaching values at 28 days. The reason behind the phosphate compounds’ inability to effectively immobilize Zn may be its intermediate dissociation. Moreover, leaching by MgO and CaO creates an alkaline environment for the immobilization of metals [38].

In addition, high cadmium leaching was observed for all three phosphate compounds, with slightly better performance for Na_2_HPO_4_, which exceeded the inert waste limit of 2 mg/kg, as illustrated in Figure 3e. OPC, on the other hand, showed excellent performance, with cadmium leaching below the inert waste limit across all conditions. Likewise, MgO and CaO showed high cadmium leaching, specifically at 2000 ppm after 28 days. The reason behind Na_2_HPO_4_’s high performance is related to the insoluble cadmium phosphate complexes that enable immobilization. The high leaching of cadmium is due to the formation of unstable complexes or those that might be soluble under certain conditions. Meanwhile, the variations among the three phosphate groups are due to fluctuations in their pH and long-term exposure, which leads to the strong immobilization of cadmium. MgO and CaO also rely on increasing the pH to immobilize cadmium through hydroxide precipitation [39]. The reason for this might be the reduction in pH with the passage of time, which further leads to cadmium hydroxides and increases cadmium’s stability [40].

Manganese leaching was also high with the three phosphate compounds, which suggests that manganese’s complexation is weak or unstable under these experimental conditions. Manganese has a different nature compared to other metals as it does not form strong insoluble complexes with phosphates at a high pH, as illustrated in Figure 3f. OPC shows low leaching due to its ability to generate a highly alkaline environment, which promotes manganese hydroxides, with low solubility in these conditions. MgO and CaO, on the other hand, perform better, but they still require a longer period of time to achieve manganese leaching; this helps to raise the pH, which further assists in the precipitation of Mn (OH)_2,_ but their immobilization capacity weakens with time [41]. The leaching increases with the passage of time, especially for Na_3_PO_4_, NaH_2_PO_4_, MgO, and CaO. This highlights the initial immobilization mechanisms, which may degrade or release more manganese into the environment.

### 3.3. Post-Experimental Characterization of Multi-Metal-Contaminated Soils After 28 Days

#### 3.3.1. X-Ray Diffraction Analysis

X-ray diffraction (XRD) is an effective analytical method employed for the investigation of the crystal structures of materials. This technique enables the identification and characterization of several crystalline structures and phases within a sample by comparing the diffraction pattern with established reference patterns. Thus, in this study, the XRD patterns and the common peaks at 2θ were examined. The majority of the samples, irrespective of the binder employed, displayed a visible peak within the range of 25° to 30°, as shown below (Figure 3). These peaks are likely analogous to quartz, which is a prevalent mineral present in soil, demonstrating that the fundamental mineral composition of the soil remained intact even after treatment.

Metals such as Cu, Ni, Zn, Pb, Cd, and Mn in contaminated soil often form metal oxide or hydroxide phases. These exhibit specific diffraction peaks in the XRD spectra at characteristic 2θ angles. Peaks at around 20–25° 2θ represent lead oxide (PbO), while the peaks at around 31.7°, 34.4°, and 36.2° 2θ represent zinc oxide (ZnO). On the other hand, the peaks formed at around 21.7° and 26.5° reflect the presence of cadmium hydroxide Cd(OH_2_).

The spectra for Na_3_PO_4_, Na_3_HPO_4_, and NaH_2_PO_4_ revealed well-defined peaks that were less prominent in the treatments involving MgO, CaO, or OPC. The distinct peaks observed suggest the development of particular mineral phases, predominantly composed of phosphates. These phases may be associated with the immobilization of metals via precipitation as metal–phosphate complexes, as reported in [42]. Additionally, the peaks in the OPC-treated soil were larger and less defined, indicating the production of weakly crystalline calcium silicate hydrates (C-S-H) or amorphous phases, which are characteristic of OPC. Similar results were also found in [43]. The complex and less ordered nature of OPC hydration products is indicated by the absence of prominent peaks [31,44].

Additionally, when comparing the various phosphate-based binders, it was found that the specific diffraction peaks seen in these treatments indicated the development of solid mineral phases, most likely incorporating different phosphate compounds. These phases revealed the potential mechanisms behind the stabilization of heavy metals by virtue of chemisorption or precipitation as insoluble phosphate salts [45].

Moreover, all of the samples exhibited an obvious peak between 25° and 30°, irrespective of the binder employed. This particular peak is commonly associated with quartz (SiO_2_), which is a widely distributed mineral, present in various soil types due to its chemical stability and low treatment–binder interactions [46]. The persistence of this peak after the stabilization process not only suggests that quartz remains the dominant and most visible phase in the treated soil matrix, but also indicates that the original mineral composition is unchanged. These findings suggest that quartz is a persistent mineral phase that strengthens soils, even with diverse stabilizing agents; this is consistent with the results found in [47]. Quartz’s resilience protects the soil matrix while allowing the binders to interact with contaminants and other reactive soil elements [48].

Moreover, several studies have documented similar results, demonstrating that phosphate treatments were able to successfully immobilize heavy metals in polluted soils [33]. Insoluble phosphate compounds, such as pyromorphite (Pb(PO_4_)_3_Cl), have made a significant contribution to decreasing the mobility and bioavailability of heavy metals [49]. The efficiency of phosphate-based binders can be ascribed to their capacity to create durable, slowly dissolving metal–phosphate complexes, which are less susceptible to leaching in different environmental circumstances.

#### 3.3.2. Fourier Transform Infrared Spectroscopy (FTIR) Analysis

**Figure 4:**
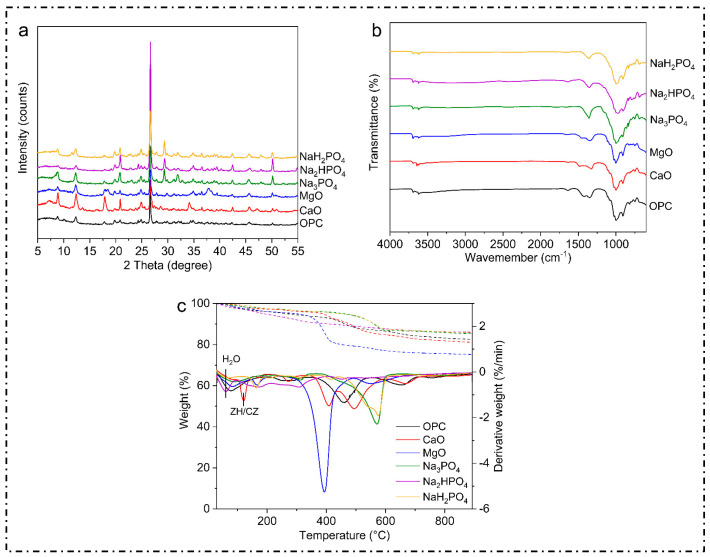
Post-experimental characterization of contaminated soils after 28 days. (**a**) explains the Xrd results of the contaminated soil after 28 days, (**b**) explains the FTIR results of the contaminated soil after 28 days, (**c**) explains the TGA/DTG results of the contaminated soil after 28 days.

The Fourier transform infrared spectroscopy (FTIR) graph compares the transmittance spectra for the multi-metal-polluted soils treated with several phosphate-based binders (NaH_2_PO_4_, Na_2_HPO_4_, Na_3_PO_4_) and traditional binders such as MgO, CaO, and OPC (Figure 4). These spectra provide information on the functional groups and molecular interactions in the treated soils. In the present study, the FTIR spectra for all samples showed a large absorption band at approximately 3400 cm^−1^ which is ascribed to O-H stretching vibrations. This indicates the existence of hydroxyl groups (-OH), which reflect hydration processes or water molecules. However, binders such as OPC, CaO, and MgO also expressed more pronounced bands, indicating the development of calcium silicate hydrates (C-S-H) and other hydrated phases, including portlandite (Ca (OH)_2_) and brucite (Mg (OH)_2_). Analogously, absorption bands were observed at around 1640 cm^−1^ (H-O-H), and the bending peaks in this particular region are mostly caused by the bending vibrations of water molecules. The consistent existence of these peaks in all samples confirms the essential function of hydration in the remediation of polluted soils. The observed differences in the phosphate-treated soils may be attributed to variations in their levels of hydration or their water retention capacities. Additionally, the phosphate compounds in the phosphorus-treated soils showed a distinct peak at 1000–1100 cm^−1^, which indicates the synthesis of metal phosphates such as Pb-p and Zn-P, which effectively bind heavy metals together. Meanwhile, the absence of peaks or the weak peaks in the MgO, CaO, and OPC spectra imply reduced phosphate interactions, which is another unique chemical property of phosphate binders in the immobilization of heavy metals. Similarly, the bands at 500–700 cm^−1^ represent M-O stretching, indicating metal ions such as Fe, Mg, and Ca.

Next, the FTIR analysis showed broad absorption bands at 3400 cm^−1^ for all samples, signifying O-H stretching vibrations, which are often connected to hydroxyl groups or water molecules. This further suggests that chemical soil treatments produce hydroxides and hydrated mineral phases through hydration reactions. In OPC-treated soils, calcium silicate hydrate phases and portlandite (Ca(OH)_2_) cause this band. On the other hand, MgO-treated soils produce brucite (Mg(OH)_2_), which aids in heavy metal immobilization by raising the pH and facilitating metal hydroxide precipitation [50]. Other researchers have also found similar O-H stretching bands in cementitious materials used for heavy metal stabilization. Moreover, the study in [51] confirms that C-S-H and brucite phases can be found in cement-based polluted soils. These phases play a significant role in the physical encapsulation and chemical binding of lead and cadmium. Similarly, H-O-H bending absorption bands at 1640 cm^−1^ are associated with the H-O-H twisting mode of water molecules, which provides water in the binder hydration process [52].

Moreover, phosphates stabilize metals, as also described in previous studies. In the study in [53], the authors explain that phosphorus is a widely used remediation agent with strong affinity for metals in soil. In line with this, the absorption bands at 1400–1500 cm^−1^ denote C-O and O-C-O stretching vibrations; these were found for the samples treated with OPC, MgO, and CaO, which have substantial carbonate absorption bonds. These bands indicate carbonation activities that may produce carbonate minerals such as calcite (CaCO_3_). Moreover, the study in [41] found that carbonating OPC- and MgO-stabilized soils reduced the leachability of lead and zinc, supporting the present study’s FTIR results. These bands in the MgO, CaO, and OPC-treated soils indicate metal hydroxides and oxides. These substances create metal precipitation sites and change the soil pH to create insoluble metal alloys, immobilizing metals [54].

#### 3.3.3. Thermogravimetric Analysis (TGA) Analysis

The current study also obtained TGA and DTG curves for the metal-contaminated soils treated with the phosphate-based and regular binders, including MgO, CaO, and OPC, as illustrated in Figure 4. TGA determines the weight loss caused by temperature, while DTG measures the pace of weight loss. A detailed analysis of the results indicates that the initial weight loss at up to 200 °C was mainly due to the evaporation of physically adsorbed water and also the dehydration process between loosely bound moisture particles. This loss was consistent across all the binders, which indicates that the treated soils contained similar amounts of free and weakly bound water. The same outcome occurred for the MgO-treated soil, with the weight loss signified by the DTG peaks at 400–500 °C; this assists in transforming magnesium hydroxide (brucite, Mg(OH)_2_) to MgO and water, which decompose at this temperature.

Furthermore, the results suggest that thermally stable phosphate complexes have the ability to bind metals even at moderate temperatures and without any structural changes. On the other hand, DTG (Derivative Thermogravimetry) measures the rate of weight loss (how quickly the weight decreases) at each temperature. The dotted lines correspond to the right y-axis (%/min) and show peaks at the temperatures where weight loss occurs most rapidly. Whereas, the peaks in the dotted line indicate specific thermal events: for instance A peak at at ~400–500 °C for MgO means that this is the temperature range where decomposition is the most intense. While, weight loss at 600 °C or higher indicates that the treated soils are thermally stable. This stability suggests that primary phase transitions and decomposition occur at lower absolute temperatures. The observed peaks indicate the occurrence of heat processes such as hydrate dehydration and carbonate or metal–phosphate complex breakdown. Likewise, the MgO-treated soil’s large increase at 400–500 °C reflects brucite decomposition. Blue dotted line shows a large and sharp peak around 400–500 °C. After this peak there is no sharp significant peak which indicate stability and high temperature. Moreover, Orange dotted line is represented by NaH_2_PO_4_, further exhibits multiple smaller peaks at lower temperature of (~100–300 °C).However, these peaks are not as sharp and intense as MgO’s. Furthermore, solid lines in the graph represent how much of the material’s weight is remaining (y-axis: Weight (%)) as the temperature increases (x-axis: Temperature (°C)). ZH/CZ amid solid lines refers to specific hydrated phases (like zeolites or calcium hydroxides) in the material. These phases lose water or decompose at specific temperatures (indicated near 200–400 °C in the graph).

On the other hand, Figure 4 presents the weight loss for each temperature, suggesting that the most abundant hydrate appeared with OPC. The hydrated C-S-H might provide substantial strength and promote the adsorption and encapsulation of toxic metals, as reported in [55]. Moreover, the reduction in mass loss in this study indicates that the metals were immobilized more effectively by the binder, rendering them more stable in the soil. Furthermore, CaCO_3_ appeared to be the predominant hydration product in this sample, which was consistent with the FTIR results. It is proposed that CaCO_3_ could sequester toxic metals, and CaO could promote the precipitation of toxic metals by increasing the pH of the solidified bodies, which is also consistent with the study in [30].

The formation of stable metal–phosphate compounds may explain the large peaks found for phosphate-treated soils, which showed more constant thermal behavior; this is consistent with the results in [24]. Likewise, another study [56] obtained the same results; namely, the authors found that phosphate treatments led to the formation of lasting lead, cadmium, and arsenic compounds. These compounds resist thermal degradation well. In our work, the phosphate-treated soils experienced lower weight loss according to the TGA/DTG curves, indicating thermal stability and efficient immobilization.

## 4. Conclusions

The present study provides evidence that ordinary Portland cement consistently outperforms other binding agents in immobilizing heavy metals, including Mn, Cd, Cu, Ni, and Zn. This superiority of OPC might be attributed to its strong alkalinity and the occurrence of pozzolanic reactions, which simplify the physical encapsulation and formation of stable hydroxide complexes. Moreover, the effectiveness of phosphate-based binders with regard to heavy metal immobilization is limited, particularly regarding Na_3_PO_4_ and NaH_3_PO_4_, which display higher leaching owing to the development of soluble metal–phosphate complexes. Binders should be selected with caution, bearing in mind the balance between the financial cost, the environmental consequences, and the particular requirements of the given project.

## Data Availability

The data presented in this study are available on request from the corresponding author. The data are not publicly available due to privacy.
